# Right ventricular remodelling in pulmonary arterial hypertension predicts treatment response

**DOI:** 10.1136/heartjnl-2021-320733

**Published:** 2022-05-05

**Authors:** Ze Ming Goh, Nithin Balasubramanian, Samer Alabed, Krit Dwivedi, Yousef Shahin, Alexander M K Rothman, Pankaj Garg, Allan Lawrie, David Capener, A A Roger Thompson, Faisal Alandejani, Jim M Wild, Christopher S Johns, Robert A Lewis, Rebecca Gosling, Michael Sharkey, Robin Condliffe, David G Kiely, Andrew J Swift

**Affiliations:** 1 Department of Infection, Immunity and Cardiovascular Disease, The University of Sheffield, Sheffield, UK; 2 Radiology Department, Sheffield Teaching Hospitals NHS Trust, Sheffield, UK; 3 INSIGNEO, Institute of Insilico Medicine, Sheffield, UK; 4 Norwich Medical School, University of East Anglia, Norwich, UK; 5 Sheffield Pulmonary Vascular Disease Unit, Sheffield Teaching Hospitals NHS Foundation Trust, Sheffield, UK

**Keywords:** Magnetic Resonance Imaging, Pulmonary Arterial Hypertension, Heart Failure

## Abstract

**Objectives:**

To determine the prognostic value of patterns of right ventricular adaptation in patients with pulmonary arterial hypertension (PAH), assessed using cardiac magnetic resonance (CMR) imaging at baseline and follow-up.

**Methods:**

Patients attending the Sheffield Pulmonary Vascular Disease Unit with suspected pulmonary hypertension were recruited into the ASPIRE (Assessing the Spectrum of Pulmonary hypertension Identified at a REferral Centre) Registry. With exclusion of congenital heart disease, consecutive patients with PAH were followed up until the date of census or death. Right ventricular end-systolic volume index adjusted for age and sex and ventricular mass index were used to categorise patients into four different volume/mass groups: low-volume-low-mass, low-volume-high-mass, high-volume-low-mass and high-volume-high-mass. The prognostic value of the groups was assessed with one-way analysis of variance and Kaplan-Meier plots. Transition of the groups was studied.

**Results:**

A total of 505 patients with PAH were identified, 239 (47.3%) of whom have died at follow-up (median 4.85 years, IQR 4.05). The mean age of the patients was 59±16 and 161 (32.7%) were male. Low-volume-low-mass was associated with CMR and right heart catheterisation metrics predictive of improved prognosis. There were 124 patients who underwent follow-up CMR (median 1.11 years, IQR 0.78). At both baseline and follow-up, the high-volume-low-mass group had worse prognosis than the low-volume-low-mass group (p<0.001). With PAH therapy, 73.5% of low-volume-low-mass patients remained in this group, whereas only 17.4% of high-volume-low-mass patients transitioned into low-volume-low-mass.

**Conclusions:**

Right ventricular adaptation assessed using CMR has prognostic value in patients with PAH. Patients with maladaptive remodelling (high-volume-low-mass) are at high risk of treatment failure.

## Introduction

Pulmonary arterial hypertension (PAH) is a life-shortening condition characterised by a vasculopathy affecting pulmonary arterioles, resulting in increased afterload and without treatment right ventricular (RV) failure.^1–3^ Assessment of disease severity and prognosis is essential in selecting treatment options, timing of lung transplantation and counselling patients.^4^

Cardiac magnetic resonance (CMR) provides an accurate and reproducible assessment of ventricular morphology and function.^5–12^ Using CMR, previous studies have assessed the prognostic value of the ratio of RV mass to volume in individuals with PAH.^2 13^ Patients with low RV mass to volume ratio (eccentric hypertrophy) have been suggested to have more severe disease than patients with high RV mass to volume ratio (concentric hypertrophy) in terms of clinical presentation, haemodynamic status and survival.^2 13^ However, a low RV mass to volume ratio may be suboptimal in identifying at-risk patients because it indicates either a normal or a dilated RV due to eccentric hypertrophy.^2^ A more recent and detailed approach to assessing RV morphology in patients with PAH using CMR is through RV volume/mass grouping, in which the RV morphology is categorised based on RV volume and mass threshold values.^14^

The aims of this study were to characterise patients with PAH based on patterns of RV adaptation using RV volume/mass grouping, assess whether RV adaptation prior to treatment predicted the likelihood of reverse remodelling following PAH therapy and to assess the impact of remodelling on prognosis.

## Methods

### Patients

Consecutive patients who underwent CMR and were diagnosed with PAH from the ASPIRE (Assessing the Spectrum of Pulmonary Hypertension Identified at a REferral Centre) Registry between 12 May 2009 and 1 February 2015 were included in the baseline cohort. Entry criteria for the registry included patients attending the Sheffield Pulmonary Vascular Disease Unit with suspected pulmonary hypertension. Patients were given the opportunity to opt out of the registry. Patients were evaluated with lung function, exercise testing, high-resolution CT, CT pulmonary angiography, CMR and right heart catheterisation (RHC), as previously described.^15^ Patients with PAH associated with congenital heart disease were excluded from the study.

Patients from the baseline cohort who had a follow-up CMR assessment were included in the follow-up cohort. Treatment regimen and follow-up were based on the European Society of Cardiology (ESC)/European Respiratory Society (ERS) guidelines^16^ and in accordance with the UK commissioning policy for treatment of PAH. Patients were treated with monotherapy, combination therapy or iloprost.

### CMR acquisition

CMR images were acquired in supine position using a GE HDx (GE Healthcare, Milwaukee, Wisconsin) whole-body scanner at 1.5T with an eight-channel cardiac coil. A stack of short-axis cine images with a slice thickness of 8 mm (2 mm interslice gap) covering both ventricles from the base to the apex were produced. A cardiac gated multislice balanced steady-state free precession (SSFP) sequence and retrospective ECG gating were used. End-systole was defined as the smallest cavity area, while end-diastole was defined as the largest cavity area or the first cine phase of the R-wave triggered acquisition.

### Image analysis

**Figure 1:**
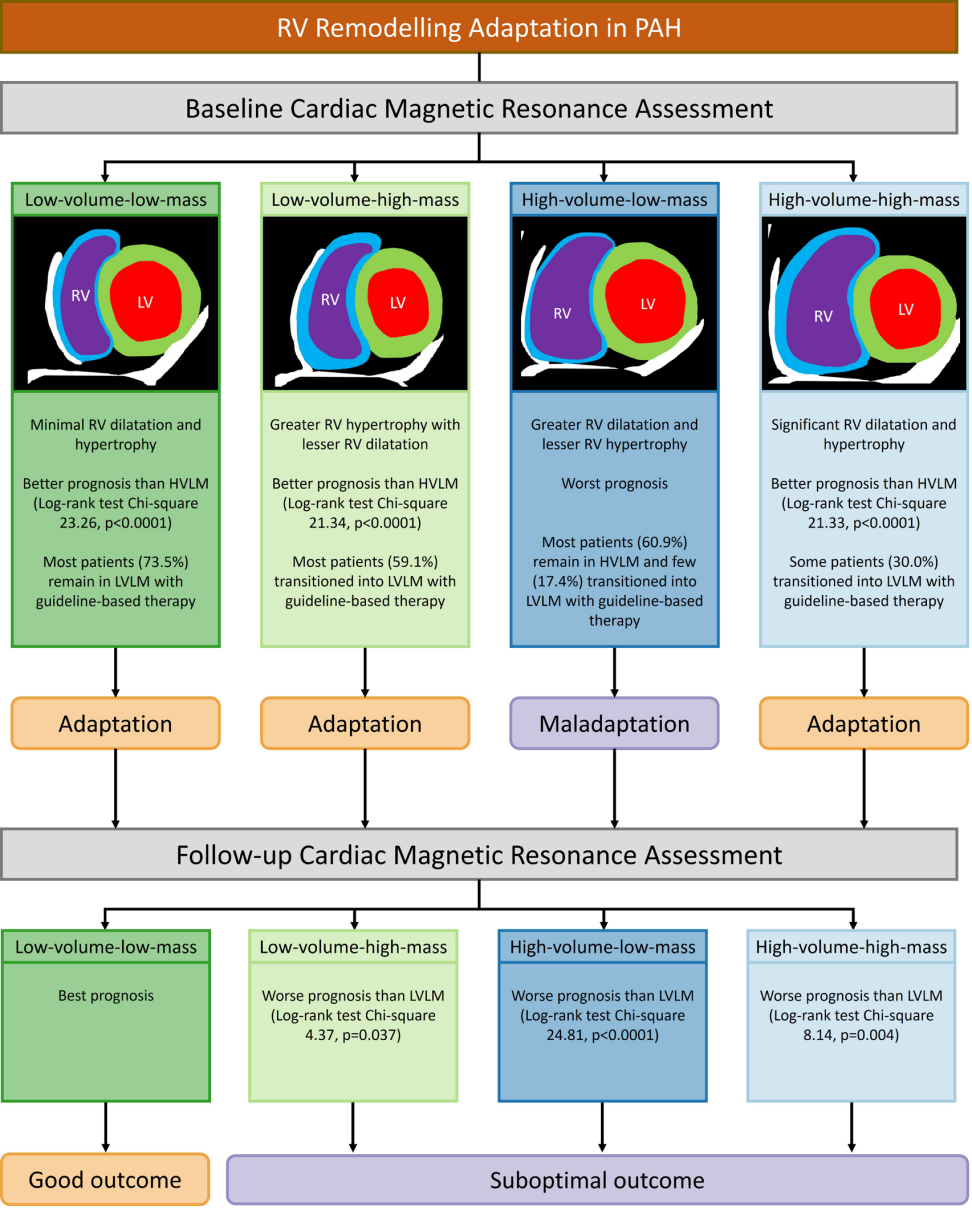
Summary figure: RV remodelling adaptation in PAH. HVLM, high-volume-low-mass; LV, left ventricular; LVLM, low-volume-low-mass; PAH, pulmonary arterial hypertension; RV, right ventricular.

Image analysis was carried out on a GE Advantage Workstation V.4.1. CMR readers were blinded to all clinical and imaging data. RV volume parameters such as RV end-diastolic volume (RVEDV), RV end-systolic volume (RVESV), left ventricular end-diastolic volume (LVEDV) and left ventricular end-systolic volume (LVESV) were obtained by manually tracing the right and left endocardial and epicardial surfaces on short-axis cine images, using proprietary MR Workstation software. RVEDV, RVESV, LVEDV and LVESV were corrected for body surface area, adjusted for sex and age, and presented as percentage predicted (RVEDVI_%pred_, RVESVI_%pred_, LVEDVI_%pred_ and LVESVI_%pred_) according to the work of Maceira *et al*.^17 18^ RV end-diastolic mass (RVEDM) and left ventricular end-diastolic mass (LVEDM) were derived. The interventricular septum was measured as part of the left ventricle (LV). Ventricular mass index (VMI) was calculated by dividing the RVEDM with the LVEDM. Figure 1 illustrates the CMR images with different RVESVI_%pred_ and VMI values. RV ejection fraction (RVEF), LV ejection fraction (LVEF), and RV and LV stroke volume were derived from the end-diastolic and end-systolic volume. RVEF and LVEF were adjusted for sex and age and presented as percentage predicted (RVEF_%pred_ and LVEF_%pred_) according to previous studies.^17 19^ Right atrial (RA) area was measured on four-chamber cine images at the phase of maximal area.

### Right heart catheterisation

RHC was performed via the internal jugular vein with a Swan-Ganz catheter. The diagnostic criteria for PAH at the time of RHC were mean pulmonary arterial pressure (mPAP) ≥25 mm Hg at rest, pulmonary arterial wedge pressure (PAWP) of ≤15 mm Hg and pulmonary vascular resistance (PVR) >3 Wood units.^16^ PVR was calculated using the following formula: PVR=(mPAP−PAWP)/cardiac output (CO). The thermodilution technique was used to obtain the values of CO.

### RV-pulmonary arterial coupling measurements

Pulmonary arterial elastance (Ea) was estimated by dividing the difference between mPAP and PAWP by LV stroke volume (LVSV) index. RV elastance (Ees) was estimated as the mPAP divided by the RV end-systolic volume index (RVESVI). RV-pulmonary arterial coupling metric (Ees:Ea) was defined as the ratio of Ees to Ea and was calculated using the following equation: (mPAP/RVESV)/([mPAP−PAWP]/LVSV).^20^ Using only the CMR measurements, a non-invasive RV-pulmonary arterial coupling metric (CMR Ees:Ea) was derived by dividing the LVSV by the RVESV.^21^

### Statistics

#### Baseline assessment

IBM SPSS Statistics V.26 was used for statistical analysis. The follow-up period was defined as the interval from the day of baseline CMR assessment to all-cause death or 28 October 2019. RVEDVI_%pred_, RVESVI_%pred_ and VMI were used to derive RV volume/mass variables such as RVEDVI_%pred_:VMI and RVESVI_%pred_:VMI. Cox proportional hazards regression was used to identify variables that were prognostic in the subgroup of incident patients. Univariate Cox proportional hazards regression analysis was used to assess the prognostic values of RVEDVI_%pred_, RVESVI_%pred_, VMI, RVEDVI_%pred_:VMI and RVESVI_%pred_:VMI. Bivariate analysis with an enter approach was run for RVESVI_%pred_ and VMI (online supplemental file 1). Variable scaling was performed to allow direct comparison of HR.

10.1136/heartjnl-2021-320733.supp1 Supplementary data

The patients were divided into four different volume/mass groups using an RVESVI_%pred_ threshold of 227% and a VMI threshold of 0.53 according to Lewis *et al* 7 and Goh *et al*.^14^ The groups were as follows: low RVESVI_%pred_ and low VMI (low-volume-low-mass), low RVESVI_%pred_ and high VMI (low-volume-high-mass), high RVESVI_%pred_ and low VMI (high-volume-low-mass), as well as high RVESVI_%pred_ and low VMI (high-volume-high-mass). RVESVI_%pred_ value was used as the threshold instead of RVEDVI_%pred_ due to its higher prognostic value.^1 4^ One-way analysis of variance (ANOVA) or χ^2^ test was used to assess the demographic, CMR, RHC and coupling variables. Differences were considered statistically significant if p<0.05 after Bonferroni correction.

Survival analysis was performed in the subgroup of incident patients. Kaplan-Meier plots were constructed and log-rank (Mantel-Cox) test was used to compare the prognoses of the volume/mass groups.

#### Follow-up assessment

Patients included in the follow-up cohort were divided into different volume/mass groups as previously described.^14^ An alluvial graph was created to demonstrate the transition in volume/mass groups from baseline to follow-up assessment. Kaplan-Meier plots were constructed and the log-rank (Mantel-Cox) test was used to compare the prognoses of the groups at follow-up. Differences were considered statistically significant if p<0.05 after correcting for false discovery rate. Multivariate Cox proportional hazards regression analysis with forward approach was used to identify demographic prognostic indicators that were independent of volume/mass groups (online supplemental file 1).

### Patient and public involvement

The Sheffield Hospital Cardiovascular Patient Panel was consulted on the importance of CMR in patient assessment and this helped to design the study to identify at-risk patients who may benefit from intensified therapy.

## Results

### Baseline assessment

#### Patients

A total of 505 consecutive individuals with PAH were identified in the baseline cohort, consisting of 362 incident treatment-naïve patients and 143 prevalent patients on PAH treatment. Within the follow-up period (median 4.85 years, IQR 4.05), 239 (47.3%) patients died. The mean age of the patients was 59±16 and 161 (32.7%) were male. Clinical subtypes included idiopathic PAH (n=256, 50.7%), connective tissue disease-associated PAH (n=192, 38.0%) and other subtypes (n=57, 11.3%). Complete RV mass, volume and RA area data were available for 493 patients. We excluded 12 patients (2.4%) due to missing data. The patients were divided into different volume/mass groups as follows: 181 low-volume-low-mass, 78 low-volume-high-mass, 74 high-volume-low-mass and 160 high-volume-high-mass. Table 1 shows the demographics and comparisons of the volume/mass groups. There were similar proportions of patients from each group who received the same treatment regimen, except in the low-volume-low-mass group, where more patients were treated with monotherapy and fewer treated with iloprost, compared with the high-volume-high-mass group.

**Table 1:** Demographics and comparison of different volume/mass groups at baseline

	All groups (n=493)	LVLM (n=181)	LVHM (n=78)	HVLM (n=74)	HVHM (n=160)
Demographics					
Age (years)	59 (16)	58*† (15)	50*§¶ (17)	67†§** (12)	59¶** (16)
Sex, male/female, n (male %)	161/332 (33)	56/125 (31)	25/53 (32)	23/51 (31)	57/103 (36)
WHO functional class, n (%)					
I	4 (0.8)	1 (0.6)	3 (3.8)	0 (0.0)	0 (0.0)
II	38 (7.7)	18 (10.1)	4 (5.1)	2 (2.7)	14 (8.8)
III	384 (78.2)	152‡ (84.9)	65 (83.3)	53 (71.6)	114‡ (71.3)
IV	65 (13.2)	8†‡ (4.5)	6§ (7.7)	19†§ (25.7)	32‡ (20.0)
PAH subtype, n (%)					
IPAH	252 (51.1)	67*‡ (37.0)	45* (57.7)	34** (45.9)	106‡** (66.3)
PAH-CTD	186 (37.7)	95*‡ (52.5)	18*§ (23.1)	36§** (48.6)	37‡** (23.1)
Other subtypes	55 (11.2)	19 (10.5)	15 (19.2)	4 (5.4)	17 (10.6)
Treatment regimen, n (%)					
Monotherapy	140 (28.7)	62‡ (34.8)	23 (29.9)	21 (28.4)	34‡ (21.5)
Combination	256 (52.6)	94 (52.8)	42 (54.5)	39 (52.7)	81 (51.3)
Iloprost	91 (18.7)	22‡ (12.4)	12 (15.6)	14 (18.9)	43‡ (27.2)
Incremental shuttle walking test distance (m)	219.07 (185.31)	247.48† (202.99)	291.79§¶ (209.42)	125.40†§ (135.68)	192.02¶(144.89)
Right heart catheterisation					
Mean right atrial pressure (mm Hg)	10 (6)	7†‡ (4)	9§¶ (5)	12†§ (5)	13‡¶ (6)
Mean pulmonary arterial pressure (mm Hg)	48 (13)	40*†‡ (11)	53*§ (14)	47†§** (11)	55‡** (9)
Pulmonary arterial wedge pressure (mm Hg)	10 (3)	10 (3)	10 (3)	11 (3)	10 (4)
Cardiac output (L/min)	5.0 (1.7)	5.7*†‡ (1.7)	4.9* (1.3)	4.8† (1.9)	4.4‡ (1.6)
Cardiac index (L/min/m^2^)	2.8 (0.9)	3.2*†‡ (0.9)	2.7* (0.7)	2.7† (1.0)	2.5‡ (0.9)
Pulmonary vascular resistance (dyn/s)	687 (394)	462*†‡ (268)	770* (391)	701†** (372)	906‡** (392)
Mixed venous oxygen saturation (%)	63 (9)	68*†‡ (7)	64*¶ (8)	61† (10)	59‡¶ (9)
CMR imaging					
RA area (cm^2^)	26.82 (10.35)	21.25*†‡ (7.00)	24.60*§¶ (8.08)	29.63†§ (8.77)	32.89‡¶ (11.42)
RVEDVI_%pred_ (%)	126.25 (44.28)	94.57†‡ (19.86)	99.23§¶ (19.90)	152.33†§ (31.12)	163.21‡¶ (42.32)
RVESVI_%pred_ (%)	247.66 (122.74)	147.93†‡ (41.84)	172.56§¶ (41.71)	326.79†§** (84.22)	360.49‡¶** (103.55)
RVEF_%pred_ (%)	56.80 (20.83)	72.11*†‡ (16.49)	61.36*§¶ (16.65)	48.24†§** (14.33)	41.23‡¶** (15.85)
RVSVI_%pred_ (%)	68.90 (29.60)	69.02 (21.44)	61.71§ (21.58)	74.99§ (30.69)	69.46 (38.59)
LVEDVI_%pred_ (%)	70.41 (21.89)	77.75*‡ (21.27)	63.37*§ (18.38)	77.41§** (24.13)	62.35‡** (19.14)
LVESVI_%pred_ (%)	72.65 (33.30)	71.30† (31.69)	62.87§ (27.47)	85.01†§ (39.86)	73.31 (32.92)
LVEF_%pred_ (%)	98.36 (15.48)	104.40†‡ (12.83)	100.51¶ (15.02)	95.31† (16.65)	91.89‡¶ (15.11)
LVSVI_%pred_ (%)	50.71 (26.57)	61.59*‡ (27.45)	41.05*§ (18.33)	61.21§** (30.78)	38.31‡** (18.70)
RVEDM (g)	53.25 (32.20)	28.64*†‡ (11.36)	73.49*§ (30.14)	37.74†§** (13.31)	78.40‡** (30.03)
LVEDM (g)	90.38 (24.76)	88.41 (24.85)	91.38 (26.91)	95.46 (26.64)	89.75 (22.46)
VMI	0.59 (0.32)	0.33*‡ (0.10)	0.81*§¶ (0.26)	0.40§** (0.09)	0.88‡¶ (0.27)
RVEDVI_%pred_:VMI	262.62 (133.78)	317.62*†‡ (127.63)	132.28*§¶ (42.42)	400.71†§** (114.91)	200.09‡¶** (73.58)
RVESVI_%pred_:VMI	487.44 (256.02)	490.73*† (203.49)	228.76*§¶ (77.74)	853.27†§** (242.33)	440.64¶** (169.49)
RV-pulmonary arterial coupling metrics					
Ea (mm Hg/mL/m^2^)	2.07 (1.42)	1.22*‡ (0.82)	2.77*§ (1.48)	1.63§** (1.12)	2.92‡** (1.41)
Ees (mm Hg/mL/m^2^)	0.95 (0.43)	1.20†‡ (0.45)	1.20§¶ (0.39)	0.70†§ (0.23)	0.69‡¶ (0.21)
Ees:Ea ratio	0.80 (0.85)	1.40*†‡ (1.05)	0.58*¶ (0.45)	0.60†** (0.40)	0.28‡¶** (0.16)
CMR Ees:Ea ratio	0.58 (0.46)	0.93*†‡ (0.49)	0.51*¶ (0.34)	0.44†** (0.27)	0.23‡¶** (0.13)

Values are presented as mean (SD) unless otherwise stated.

Treatment data are not available for 6 patients.

*P<0.05 after Bonferroni correction when LVLM was compared with LVHM.

†P<0.05 after Bonferroni correction when LVLM was compared with HVLM.

‡P<0.05 after Bonferroni correction when LVLM was compared with HVHM.

§P<0.05 after Bonferroni correction when LVHM was compared with HVLM.

¶P<0.05 after Bonferroni correction when LVHM was compared with HVHM.

**P<0.05 after Bonferroni correction when HVLM was compared with HVHM.

CMR, cardiac magnetic resonance; Ea, arterial load; Ees, right ventricular elastance; HVHM, high-volume-high-mass; HVLM, high-volume-low-mass; IPAH, idiopathic pulmonary arterial hypertension; LVEDM, left ventricular end-diastolic mass; LVEDVI_%pred_, left ventricular end-diastolic volume index percentage predicted; LVEF_%pred_, left ventricular ejection fraction percentage predicted; LVESVI_%pred_, left ventricular end-systolic volume index percentage predicted; LVHM, low-volume-high-mass; LVLM, low-volume-low-mass; LVSVI_%pred_, left ventricular stroke volume index predicted; PAH-CTD, connective tissue disease-associated pulmonary arterial hypertension; RA, right atrial; RA, right atrial area; RVEDM, right ventricular end-diastolic mass; RVEDVI_%pred_, right ventricular end-diastolic volume index percentage predicted; RVEDVI_%pred_:VMI, ratio of right ventricular end-diastolic volume index percentage predicted to ventricular mass index; RVEF_%pred_, right ventricular ejection fraction percentage predicted; RVESVI_%pred_, right ventricular end-systolic volume index percentage predicted; RVESVI_%pred_:VMI, ratio of right ventricular end-systolic volume index percentage predicted to ventricular mass index; RVSVI_%pred_, right ventricular stroke volume index percentage predicted; VMI, ventricular mass index.

#### One-way ANOVA and χ^2^

**Figure 2:**
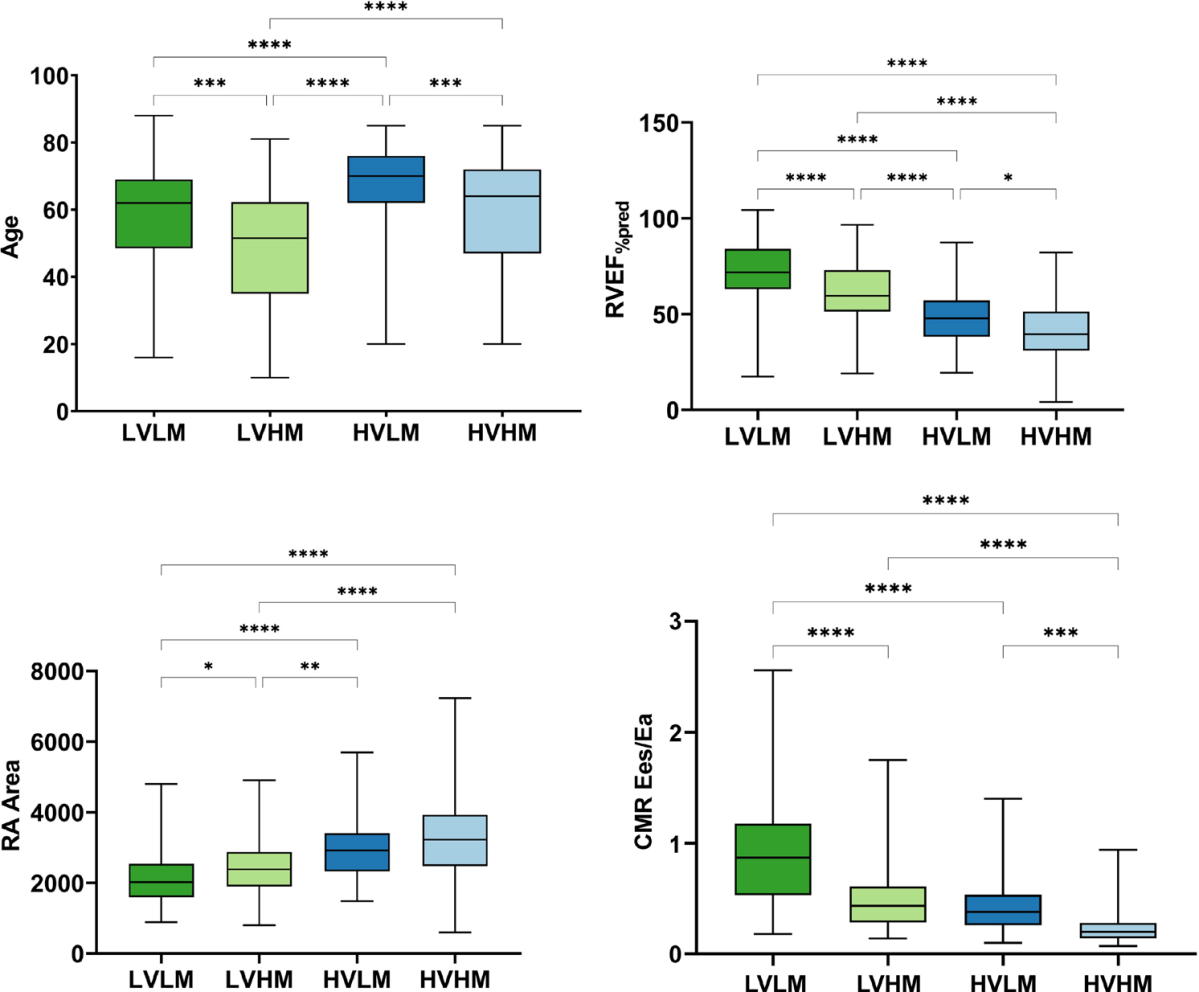
Box and whisker plot of age and CMR-derived metrics including RVEF_%pred_, RA area and CMR Ees:Ea at baseline in groups. CMR, cardiac magnetic resonance; Ea, arterial load; Ees, right ventricular elastance; HVHM, high-volume-high-mass; HVLM, high-volume-low-mass; LVHM, low-volume-high-mass; LVLM, low-volume-low-mass; RA, right atrial; RVEF_%pred_, right ventricular ejection fraction percentage predicted.*P≤0.05; **P≤0.01; ***P≤0.001; ****P≤0.0001.

**Figure 3:**
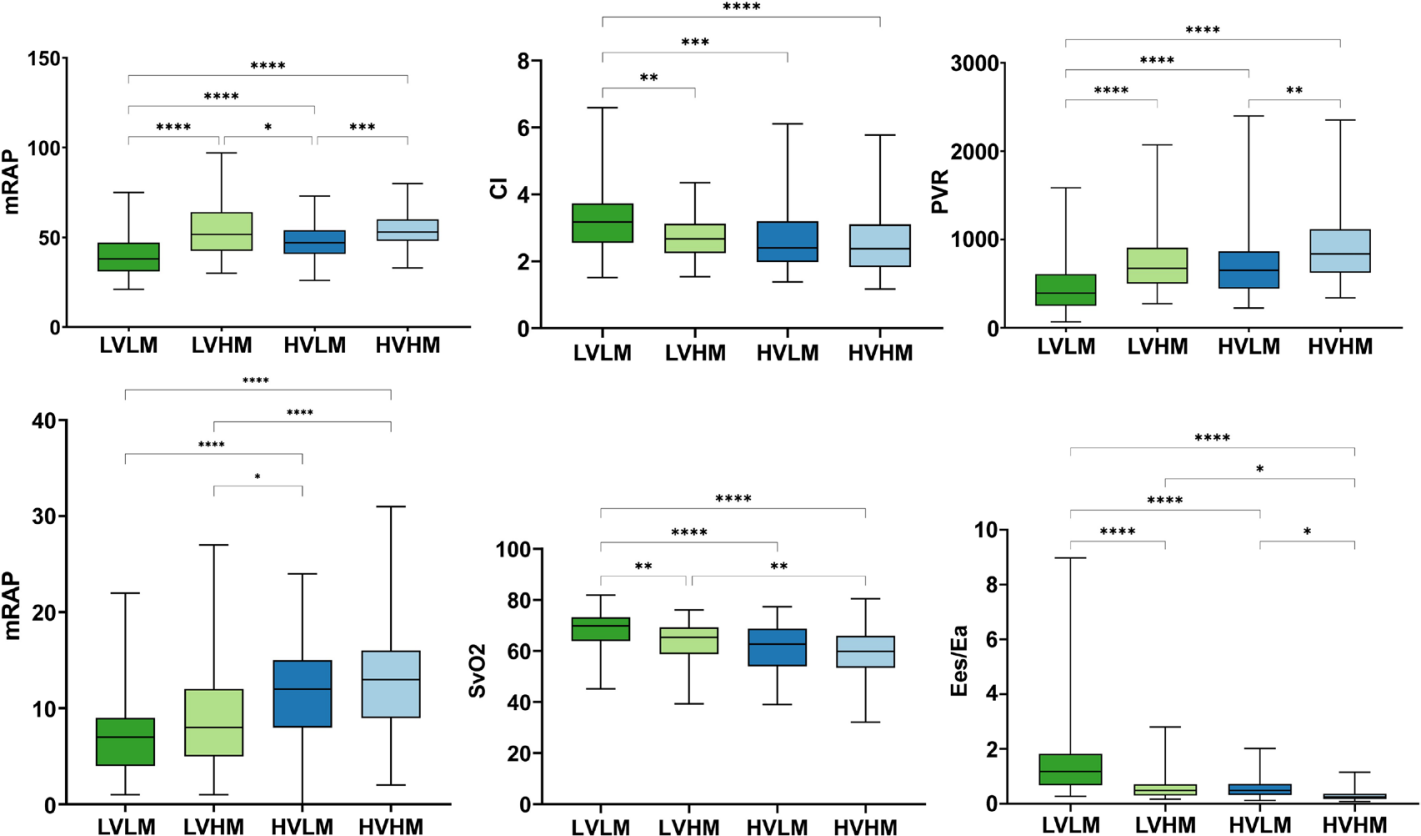
Box and whisker plot of haemodynamic metrics including mPAP, CI, PVR, mean right atrial pressure, mixed venous oxygen saturation and Ees:Ea at baseline in groups. CI, cardiac index; Ea, arterial load; Ees, right ventricular elastance; HVHM, high-volume-high-mass; HVLM, high-volume-low-mass; LVHM, low-volume-high-mass; LVLM, low-volume-low-mass; mPAP, mean pulmonary arterial pressure; PVR, pulmonary vascular resistance. *P≤0.05; **P≤0.01; ***P≤0.001; ****P≤0.0001.

Box and whisker plots were constructed to illustrate the results of one-way ANOVA (figures 2 and 3). The low-volume-low-mass group had the highest mean cardiac index (CI) (3.2±0.9), mixed venous oxygen saturation (68±7), RVEF_%pred_ (71.1±16.5), Ees:Ea (1.4±1.1) and CMR Ees:Ea (0.9±0.5), the lowest mPAP (40±11) and PVR (462±268), as well as the smallest RA area (21.2±7.0). High-volume-low-mass patients had the oldest age (67±12). The high-volume-high-mass group had the highest mean PVR (906±392) and the lowest Ees:Ea (0.3±0.2) and CMR Ees:Ea (0.2±0.1).

#### Survival analyses

A total of 362 incident patients were identified and included in the survival analyses. Within the follow-up period (median 4.85 years, IQR 4.62), 184 patients (51%) died. Table 2 displays the results of the univariate Cox proportional hazard regression analysis. Variables identified to be significant predictors of mortality in univariate Cox regression included RVESVI_%pred_ (scaled HR 1.162; 95% CI 1.013 to 1.334; p=0.032), VMI (scaled HR 0.842; 95% CI 0.719 to 0.985; p=0.032), RVEDVI_%pred_:VMI (scaled HR 1.303; 95% CI 1.127 to 1.507; p<0.001) and RVESVI_%pred_:VMI (scaled HR 1.354; 95% CI 1.194 to 1.536; p<0.001).

**Table 2:** Results of Cox regression in the subgroup of incident patients at baseline

Variables*	Univariate		Scaled univariate		P value	n
	HR	95% CI	HR	95% CI		
RVEDVI_%pred_	1.003	1.000 to 1.006	1.139	0.982 to 1.321	0.086	361
RVESVI_%pred_	1.001	1.000 to 1.002	1.162	1.013 to 1.334	0.032	361
VMI	0.598	0.374 to 0.957	0.842	0.719 to 0.985	0.032	357
RVEDVI_%pred_:VMI	1.002	1.001 to 1.003	1.303	1.127 to 1.507	<0.001	356
RVESVI_%pred_:VMI	1.001	1.001 to 1.002	1.354	1.194 to 1.536	<0.001	356
Bivariate Cox model of RVESVI_%pred_ and VMI	2.857	1.718 to 4.752	1.380	1.181 to 1.613	<0.001	356

*Congenital heart disease excluded from this group.

RVEDVI_%pred_, right ventricular end-diastolic volume index percentage predicted; RVEDVI_%pred_:VMI, ratio of right ventricular end-diastolic volume index percentage predicted to ventricular mass index; RVESVI_%pred_, right ventricular end-systolic volume index percentage predicted; RVESVI_%pred_:VMI, ratio of right ventricular end-systolic volume index percentage predicted to ventricular mass index; VMI, ventricular mass index.

On bivariate Cox regression, both RVESVI_%pred_ and VMI were identified as independent CMR predictors of death and the following model was derived: prognostic index=(RVESVI_%pred_×0.002)−(VMI×0.965) (online supplemental file 1). The bivariate Cox regression model was shown to have significant prognostic value (scaled HR 1.380; 95% CI 1.181 to 1.613; p<0.001).

#### Group comparison

**Figure 4:**
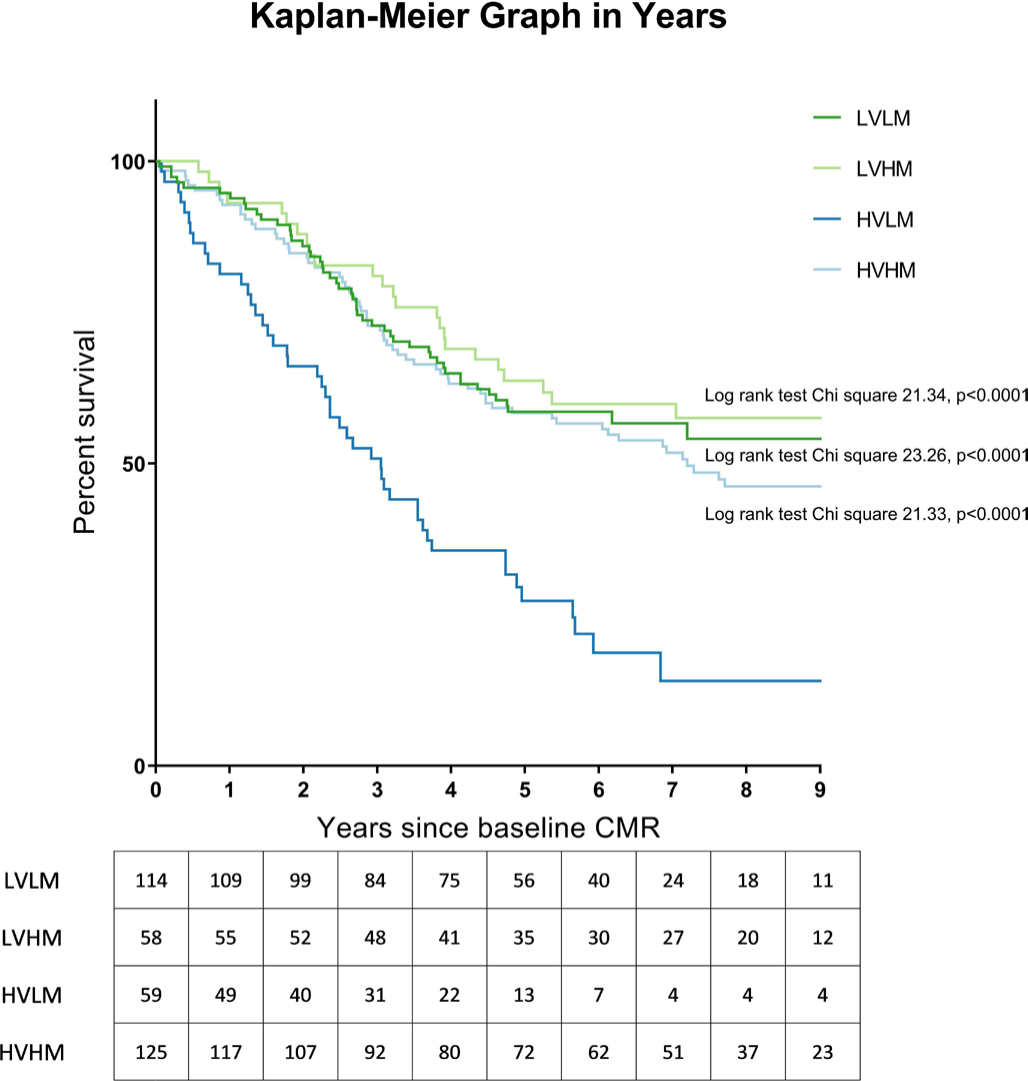
Kaplan-Meier graph illustrating survival of the subgroup of incident patients at baseline assessment, excluding patients with congenital heart disease. Log-rank test results comparing each group with HVLM are shown, with p value corrected for false discovery rate. CMR, cardiac magnetic resonance; HVHM, high-volume-high-mass; HVLM, high-volume-low-mass; LVHM, low-volume-high-mass; LVLM, low-volume-low-mass.

Figure 4 illustrates the plotted Kaplan-Meier graphs. Within the subgroup of incident patients, the prognosis of the high-volume-low-mass group was significantly worse than the low-volume-low-mass (log-rank test χ^2^ 23.26, p<0.0001), low-volume-high-mass (log-rank test χ^2^ 21.34, p<0.0001) and high-volume-high-mass group (log-rank test χ^2^ 21.33, p<0.0001) groups at baseline.

### Follow-up assessment

#### Patients

The follow-up cohort consisted of 124 (24.6%) patients out of the 505 patients from the baseline cohort who had a subsequent CMR at follow-up (median 1.11 years, IQR 0.78). Clinical subtypes included idiopathic PAH (n=64, 51.6%), connective tissue disease-associated PAH (n=48, 38.7%) and other subtypes (n=12, 9.7%). Patients were divided into different volume/mass groups as follows: 62 low-volume-low-mass, 15 low-volume-high-mass, 23 high-volume-low-mass and 24 high-volume-high-mass (Online supplemental file 1). Of the 124 patients included, 48 (38.4%) died after a further follow-up duration (median 4.36 years, IQR 2.62).

#### Group transition

**Figure 5:**
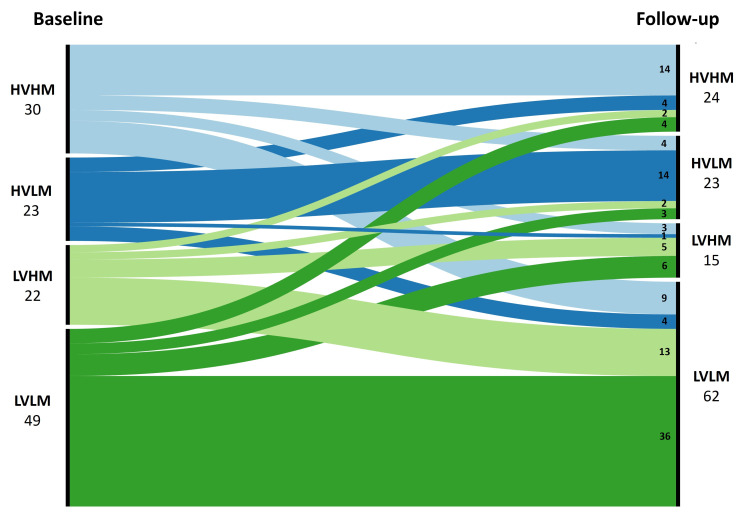
An alluvial graph demonstrating the transition of patients of each volume/mass group from baseline to follow-up with guideline-based therapy. HVHM, high-volume-high-mass; HVLM, high-volume-low-mass; LVHM, low-volume-high-mass; LVLM, low-volume-low-mass.

The transition of volume/mass group from baseline to follow-up assessment is illustrated in figure 5. Among the low-volume-low-mass patients, most remained in the same group (73.5%) at follow-up. Most low-volume-high-mass patients (59.1%) transitioned into low-volume-low-mass. More than half of the high-volume-low-mass patients (60.9%) remained in the group. Within the high-volume-high-mass group, 30.0% transitioned into low-volume-low-mass.

#### Survival analyses

**Figure 6:**
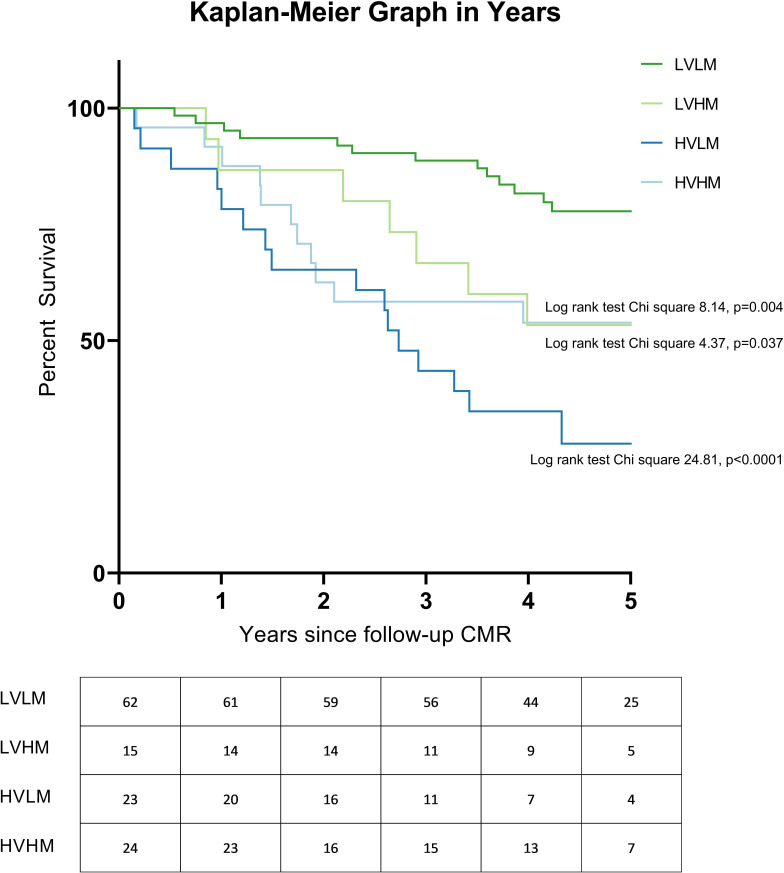
Kaplan-Meier graph illustrating survival of patients, excluding patients with congenital heart disease at follow-up. Log-rank test results comparing each group with LVLM are shown, with p value corrected for false discovery rate. The number at risk each year is presented below each plot. CMR, cardiac magnetic resonance; HVHM, high-volume-high-mass; HVLM, high-volume-low-mass; LVHM, low-volume-high-mass; LVLM, low-volume-low-mass.

Figure 6 illustrates the plotted Kaplan-Meier graphs. At follow-up assessment, patients with low-volume-low-mass had better prognosis than patients with low-volume-high-mass (log-rank test χ^2^ 4.37, p=0.037), high-volume-low-mass (log-rank test χ^2^ 24.81, p<0.0001) and high-volume-high-mass (log-rank test χ^2^ 8.14, p=0.004). On multivariate Cox regression analysis, volume/mass groups were found to be independent of age at follow-up and treatment regimen and the following model was derived: prognostic index=(age at follow-up×0.042)+(0 if low-volume-low-mass)+(1.460 if low-volume-high-mass)+(1.193 if high-volume-low-mass)+(1.377 if high-volume-high-mass) (online supplemental file 1).

## Discussion

This study has evaluated the prognostic utility of patterns of RV adaptation assessed with CMR in patients with PAH and has shown that patients with maladaptive RV remodelling, characterised by high-volume-low-mass, were associated with poor outcome and predicted a poor response to treatment. In contrast, patients with low-volume-low-mass had an excellent prognosis.

### RV remodelling

In the early stage of PAH, initial RV adaptation to elevated afterload occurs with RV function maintained through RV hypertrophy. As the disease progresses, the hypertrophic process may slow as RV dilatation progresses.^22^ A meta-analysis of 21 studies evaluating the prognostic value of CMR-derived RV volume parameters showed that for every 1 mL/m^2^ increase in RVESVI and RV end-diastolic volume index (RVEDVI), the risk of mortality increases by 1.0% and 0.6%, respectively.^1^ Using echocardiography, whereas increased RV diameter was associated with a worse prognosis, those with RV wall thickness had a better prognosis.^23^ Early compensatory increase in RV mass has been suggested to represent adaptive RV remodelling^24^ and an increased VMI is associated with lower mortality in PAH.^14^

The RV mass to volume ratio has been proposed as a better prognostic indicator than RV mass or volume alone because it allows distinction of eccentric hypertrophy (low RV mass:volume) and concentric hypertrophy (high RV mass:volume).^2 13^ In the present study, the bivariate Cox regression model of RVESVI_%pred_ and VMI was a stronger prognostic indicator than RV volume/mass variables, including RVEDVI_%pred_:VMI and RVESVI_%pred_:VMI, suggesting that a simple ratio may be suboptimal in identifying at-risk patients with PAH. In addition, the RV mass to volume ratio is limited as individuals with a low RV mass to volume ratio can indicate either a normal or dilated RV due to eccentric hypertrophy.^2^ Therefore, the present study adopted the volume/mass grouping based on RVESVI_%pred_ and VMI values to better understand the different patterns of RV adaptation in PAH and their association with RV function and survival rate. Figure 1 illustrates the different types of RV adaptation in PAH and their associated outcomes.

### RV reverse remodelling with successful interventions

RV reverse remodelling, characterised by reduction in RV mass and volume, is associated with successful interventions through pharmacological treatments, pulmonary endarterectomy or lung transplantation.^25^ Echocardiographic evidence of RV reverse remodelling has been shown to be a stronger prognostic indicator than functional and haemodynamic parameters.^25^

### RV volume/mass phenotypes: group comparison

We postulated that the prognoses of the RV volume/mass groups were determined by two factors: their structural adaptivity to the increased afterload and potential to undergo RV reverse remodelling in response to treatment. The structural adaptivity of each group was evaluated through its association with previously described CMR or RHC-derived prognostic indicators.^4 22 26–30^ Meanwhile, the likelihood of reverse remodelling due to treatment was assessed by studying the transition of patients with PAH from their respective volume/mass group to the low-volume-low-mass group during follow-up.

Low-volume-low-mass patients have been suggested to undergo minimal RV remodelling and thus maintain good RV function.^14^ This is consistent with the findings of the present study that the low-volume-low-mass group had the highest mean CI, SvO_2_, RVEF_%pred_ and Ees:Ea, the lowest mPAP and PVR, and the smallest RA area. These prognostic indicators are associated with better RV function and survival.^4 22 26–28 30^ Furthermore, better prognosis was observed in low-volume-low-mass patients at both baseline^14^ and follow-up assessments. Therefore, we propose that low-volume-low-mass is the ideal RV structural adaptation in PAH and could be used as the treatment target. Additionally, we found that most low-volume-low-mass patients (73.5%) remain in this mild or adaptive RV morphology.

Low-volume-high-mass and high-volume-high-mass groups have been suggested to represent adaptive RV changes in PAH.^14^ However, in the present study, both low-volume-high-mass and high-volume-high-mass patients had significantly lower mean RVEF_%pred_, CI, SvO_2_ and Ees:Ea than low-volume-low-mass patients, suggesting that they had impaired RV function and at risk of worse clinical outcomes.^4 22 26 28 30^ Additionally, a poor prognosis was observed for groups who attained either a low-volume-high-mass or high-volume-high-mass at follow-up. The more favourable prognosis of these groups at baseline^14^ may be explained by their potential to undergo reverse RV remodelling with treatment. Following PAH therapy, a significant proportion of low-volume-high-mass (59.1%) and high-volume-high-mass (30.0%) patients transitioned into the low-volume-low-mass group, which was associated with better RV function. Therefore, in the scenario where a low-volume-high-mass or high-volume-high-mass patient remains in their volume/mass group despite initial treatment, physicians should consider escalating treatment regimen to achieve reverse RV remodelling, or where the patient is on maximal therapy consider, where eligible, lung transplantation.

High-volume-low-mass has been recognised to be a maladaptation in PAH and is associated with poor prognosis.^14^ The current study has demonstrated consistent findings, that high-volume-low-mass patients had significantly lower CI, SvO_2_, RVEF_%pred_ and Ees:Ea, higher mPAP and PVR, as well as larger RA area than the low-volume-low-mass group. Additionally, high-volume-low-mass patients had a poor prognosis at both baseline and follow-up assessments. Despite PAH therapy, most high-volume-low-mass patients (60.9%) remained in the maladaptive group. Therefore, we postulate that high-volume-low-mass reflects a maladaptation to an increased afterload and with current PAH therapies are unlikely to successfully remodel.

High-volume-low-mass patients have been found to be older than other groups and this may in part explain their higher mortality.^14^ However, the present study has demonstrated that all volume/mass groups remained prognostic following adjustment for age at follow-up and treatment regimen, suggesting that all patients with inadequate RV reverse remodelling at follow-up (low-volume-high-mass, high-volume-low-mass and high-volume-high-mass) are at increased risk of adverse outcome regardless of age and treatment regimen. Furthermore, high-volume-low-mass patients received similar treatment as other groups at baseline in the present study, demonstrating that these high-risk patients were not detected using the risk stratification method of the ESC/ERS guidelines, and features of RV remodelling on CMR could be an early indicator of adverse outcomes. Besides, future studies that explore the link between age or genetics and susceptibility to RV maladaptation in patients with PAH might provide invaluable clinically relevant information and insight into the pathophysiology of the disease.

### Limitations

The study cohort consisted of patients referred to a single tertiary centre and would benefit from external validation. During the Sixth World Symposium on Pulmonary Hypertension in 2018, it was recommended to diagnose PAH using a threshold of mPAP >20 mm Hg and PVR ≥3 Wood units.^31^ However, the present registry study has adopted the previous diagnostic threshold of mPAP ≥25 mm Hg and PVR >3 Wood units. There were relatively few patients who received a follow-up CMR as it was not an obligation in the registry, making it difficult to compare the survival analyses at baseline and follow-up. Due to the nature of the study, it was not possible to evaluate the effect of different regimens on RV remodelling and clinical outcome. Further work with controlled treatment regimens would be beneficial.

## Conclusion

CMR identifies patients with PAH with low RV volume and mass who are well adapted to an increased RV afterload and have low mortality, and patients with high RV volume and low RV mass who represent a maladaptation with high mortality. Low RV volume and mass at follow-up should be used as a treatment target. Intensification of treatment should be considered in patients with high RV volume and low RV mass, possibly as early as at the time of diagnosis.

**Key messages**

**What is already known on this subject?**

- Right ventricular (RV) failure is considered the key determinant of mortality in pulmonary arterial hypertension (PAH).
- The relationship between RV dilatation and hypertrophy in PAH has been proposed as a marker of RV adaptation.

**What might this study add?**

- In patients receiving PAH treatment, reverse remodelling characterised by a reduction in RV mass and volume is associated with improved outcomes, with patients achieving low-volume-low-mass at follow-up having better long-term outcome, compared with low-volume-high-mass (log-rank test χ^2^ 4.37, p=0.037), high-volume-low-mass (log-rank test χ^2^ 24.81, p<0.001) and high-volume-high-mass (log-rank test χ^2^ 8.14, p=0.004).
- While patients with low-volume-high-mass (59.1%) and high-volume-high-mass (30.0%) have the potential to undergo RV reverse remodelling, response to treatment in patients with high-volume-low-mass is poor (17.4%).

**How might this impact on clinical practice?**

- The study aids the identification of patients with PAH and maladaptive remodelling (high-volume-low-mass) who are at high risk of treatment failure.

## Data Availability

Data are available upon reasonable request.
