# Supplementary material for: Right ventricular remodelling in pulmonary arterial hypertension predicts treatment response
Source: Heart. 2022 May 5;108(17):1392–400. doi: 10.1136/heartjnl-2021-320733 (PMC9380507; doi:10.1136/heartjnl-2021-320733)
Supplement: Supplementary data [file heartjnl-2021-320733supp001.pdf]

Supplementary Material

Baseline Assessment:

Correlations

Pearson's correlation test was used to identify CMR derived RV mass and volume variables that had a significant correlation with mPAP, PVR, mixed venous oxygen saturation (SvO2), $RVEF_{\%pred}$ and RA area (**Supplementary Table 2**).

Significant correlations were observed between mPAP with all RV mass and volume variables, VMI had the strongest correlation (r=0.525, p<0.001). PVR was significantly correlated with $RVEF_{\%pred}$ (r=-0.467, p<0.001), $RVEDVI_{\%pred}$/VMI (r=-0.400, p<0.001), $RVESVI_{\%pred}$/VMI (r=-0.197, p<0.001) and VMI (r= 0.465, p<0.001). Variables that correlated with SvO2 included $RVEDVI_{\%pred}$ (r= -0.212, p<0.001), $RVESVI_{\%pred}$ (r= -0.325, p<0.001), $RVEF_{\%pred}$ (r=0.404, p<0.001), $RVEDVI_{\%pred}$/VMI (r=0.189, p<0.001), VMI (r= -0.257, p<0.001) and RA area (r= -0.300, p<0.001).

Survival analyses

Cox regression model with an enter approach involves using all the selected variables in the derivation of the model.

Follow-up Assessment:

Transition of RV volume and mass parameters: Independent t-test

Independent unpaired t-test was used to compare the values of $RVESVI_{\%pred}$ and VMI in the alive and dead cohorts.

Among patients who survived, significant reduction was observed in mean $RVESVI_{\%pred}$ (from 212.6±122.0 to 170.0±81.3; p=0.012) and VMI (from 0.58±0.34 to 0.46±0.23; p=0.012). In contrast, there was no significant change in mean $RVESVI_{\%pred}$ or VMI for patients who died.

Survival Analyses

Cox regression model with a forward approach starts with an empty model and adds in variables one by one, while prioritising variable that gives the single best improvement.

Supplementary Table 1: Treatment regimen in incident and prevalent patients

Treatment Regimen	Incident (N=362)	Prevalent (N=142)
Monotherapy	121 (33.4%)	22 (15.5)
Combination therapy	183 (50.6%)	82 (57.7%)
Iloprost	53 (14.6%)	38 (26.8)
Not on treatment	5 (1.4%)	0 (0.0%)

Supplementary Table 2: Pearson Correlations of the variables

	mPAP		PVR		SvO_2	
Variable	r Value	p-value	r Value	p-value	r Value	p-value
$RVEDVI_{\%pred}$	0.261	<0.001	0.186	<0.001	-0.212	<0.001
$RVESVI_{\%pred}$	0.319	<0.001	0.326	<0.001	-0.325	<0.001
$RVEF_{\%pred}$	-0.393	<0.001	-0.467	<0.001	0.404	<0.001
$RVEDVI_{\%pred}$/VMI	-0.413	<0.001	-0.400	<0.001	0.189	<0.001
$RVESVI_{\%pred}$/VMI	-0.248	<0.001	-0.197	<0.001	0.001	0.984
VMI	0.525	<0.001	0.465	<0.001	-0.257	<0.001
RA Area	0.262	<0.001	0.197	<0.001	-0.300	<0.001

Supplementary Table 3: Bivariate Model of $RVESVI_{\%pred}$ and VMI

	B	SE	Wald	df	p-value	Hazard Ratio	95% CI Lower Limit	95% CI Upper Limit
$RVESVI_{\%pred}$	0.002	0.001	12.834	1	<0.001	1.002	1.001	1.003
VMI	-0.965	0.270	11.939	1	0.001	0.381	0.220	0.659

Supplementary Table 4: Demographics and comparison of different volume/mass groups at follow-up

	All Groups (N=124)	LVLM (N=62)	LVHM (N=15)	HVLM (N=23)	HVHM (N=24)
**Demographics**					
Age (years)	53 (16)	53† (15)	42§ (11)	67†§¶ (12)	48¶ (16)
Sex M/F (n, male %)	35/89 (28%)	19/43 (31%)	4/11 (27%)	7/16 (30%)	5/19 (21%)
**PAH subtype (n, %)**					
IPAH	64 (51.6%)	33 (53.2%)	9 (60.0%)	6¶ (26.1%)	16¶ (66.7%)
PAH-CTD	48 (38.7%)	22 (35.5%)	5 (33.3%)	15¶ (65.2%)	6¶ (25.0%)
Other Subtypes	12 (9.7%)	7 (11.3%)	1 (6.7%)	2 (8.7%)	2 (8.3%)
**Treatment regimen (n, %)**					
Monotherapy	23 (19.2%)	15 (25.9%)	3 (20.0%)	3 (13.0%)	2 (8.3%)
Combination	59 (49.2%)	31 (53.4%)	6 (40.0%)	11 (47.8%)	11 (45.8%)
Iloprost	38 (31.7%)	12 (20.7%)	6 (40.0%)	9 (39.1%)	11 (45.8%)

Treatment data is not available for 4 patients.

Supplementary Table 5: Multivariable Model of Volume/Mass group at follow-up and Age

	B	SE	Wald	df	p-value	Hazard Ratio	95% CI Lower Limit	95% CI Upper Limit
Age at Follow-up	0.042	0.012	12.452	1	<0.001	1.042	1.019	1.067
LVLM			16.367	3	0.001			
LVHM	1.460	0.495	8.689	1	0.003	4.305	1.631	11.362
HVLM	1.193	0.397	9.046	1	0.003	3.298	1.515	7.176
HVHM	1.377	0.407	11.464	1	0.001	3.965	1.786	8.800
